# Preparation of Organic Light-Emitting Diode Using Coal Tar Pitch, a Low-Cost Material, for Printable Devices

**DOI:** 10.1371/journal.pone.0062903

**Published:** 2013-05-07

**Authors:** Miki Yamaoka, Shun-suke Asami, Nayuta Funaki, Sho Kimura, Liao Yingjie, Takeshi Fukuda, Makoto Yamashita

**Affiliations:** 1 Department of Functional Materials Science, Faculty of Engineering, Saitama University, Saitama, Japan; 2 Department of Applied Chemistry, Faculty of Science and Engineering, Chuo University, Bunkyo-ku, Japan; University of Akron, United States of America

## Abstract

We have identified coal tar pitch, a very cheap organic material made from coal during the iron-making process, as a source from which could be obtained emissive molecules for organic light-emitting diodes. Coal tar pitch was separated by simple dissolution in organic solvent, and subsequent separation by preparative thin-layer chromatography was used to obtain emissive organic molecules. The retardation factor of preparative thin-layer chromatography played a major role in deciding the emission characteristics of the solution as photoluminescence spectra and emission-excitation matrix spectra could be controlled by modifying the solution preparation method. In addition, the device characteristics could be improved by modifying the solution preparation method. Two rounds of preparative thin-layer chromatography separation could improve the luminance of organic light-emitting diodes with coal tar pitch, indicating that less polar components are favorable for enhancing the luminance and device performance. By appropriate choice of the solvent, the photoluminescence peak wavelength of separated coal tar pitch could be shifted from 429 nm (cyclohexane) to 550 nm (chloroform), and consequently, the optical properties of the coal tar pitch solution could be easily tuned. Hence, the use of such multicomponent materials is advantageous for fine-tuning the net properties at a low cost. Furthermore, an indium tin oxide/poly(3,4-ethylenedioxythiophene):poly(styrenesulfonate)/coal tar pitch/LiF/Al system, in which the emissive layer was formed by spin-coating a tetrahydrofuran solution of coal tar pitch on the substrate, showed a luminance of 176 cd/m^2^. In addition, the emission spectrum of coal tar pitch was narrowed after the preparative thin-layer chromatography process by removing the excess emissive molecules.

## Introduction

Organic electronics is an important research field directed at the development of thin lightweight electronic devices, which play a key role in the establishment of an energy-efficient, sustainable society. Among various organic electronic thin-film devices, organic light-emitting diodes (OLEDs) are considered suitable for use in the preparation of display devices and illumination systems. The first example of organic electroluminescence device was made from crystalline anthracene [1], [2]. Later, Tang [3] found that a layered structure is suitable for efficient emission from an OLED; since then, several researchers have independently reported a number of high-performance OLEDs with transition-metal compounds for phosphorescence emission [4], polymer semiconductors for low-cost processing [5], multilayer structures for efficient emission [6], etc. That is, in the past, organic electronic devices were developed from well-designed organic molecules by using state-of-the-art synthetic organic chemistry, but at a relatively high cost. However, devices based on organic semiconductors are expensive, which restricts their widespread utility; hence, there is a strong demand for OLEDs based on low-cost materials. Herein, we report the use of coal tar pitch, an inexpensive material derived from coal during the iron-making process, in the fabrication of OLEDs.

Coal is one of the most important energy sources, and it usually contains many types of fused polycyclic aromatic compounds linked through alkyl chains [7], [8]. Coal is used not only as a fuel in thermal power plants but also as coke, a reductant for the production of steel from iron ore. Carbonization of coal affords coke along with a number of coal tar byproducts. Removal of coal gas, light oils, and volatiles (naphthalene and similar small polycyclic aromatic hydrocarbons, PAHs) from coal tar yields a black tarry residue, commonly referred to as “coal tar pitch,” which constitutes 5–10% of the consumed coal by weight. Because steel production is carried out on a large scale at the global level, coal tar pitch, an inexpensive carbon resource, is obtained in vast quantities and widely used for the production of carbon fiber and carbon electrodes. Since coal tar pitch is distilled from coal, it contains numerous low-molecular-weight π-functional components [9]. Furthermore, since coal tar pitch is a mixture of various components with diverse molecular structures, its composition and net photophysical properties can vary depending on the separation technique employed [10]–[17]. Carbon-based π-conjugated materials such as graphene and graphene oxide (GO) are being investigated for use as the key materials in high-performance organic devices, or as precursors to these materials [18]. Graphene is usually used to fabricate transparent electrodes for OLEDs because the infinite π-conjugated system results in electron-conducting characteristics without photoluminescence (PL). On the other hand, GO has PL characteristics [19]–[26] because the limited number of interconnected aromatic rings in this material can afford an appropriate size of π-conjugated system, smaller than the effective conjugation length. In fact, controlled reduction of GO has been reported to induce a gradual change in the PL properties [27]. Since the π-conjugated system in coal tar pitch is similar to that in GO, the former is considered a potential candidate for a low-cost organic material that can be used in printable electronics.

In this study, OLEDs in which the emissive layer was formed using coal tar pitch solutions in different solvents were fabricated, and the effect of the polarity of the coal tar pitch components on the device performance was investigated.

## Results and Discussion

**Figure 1:**
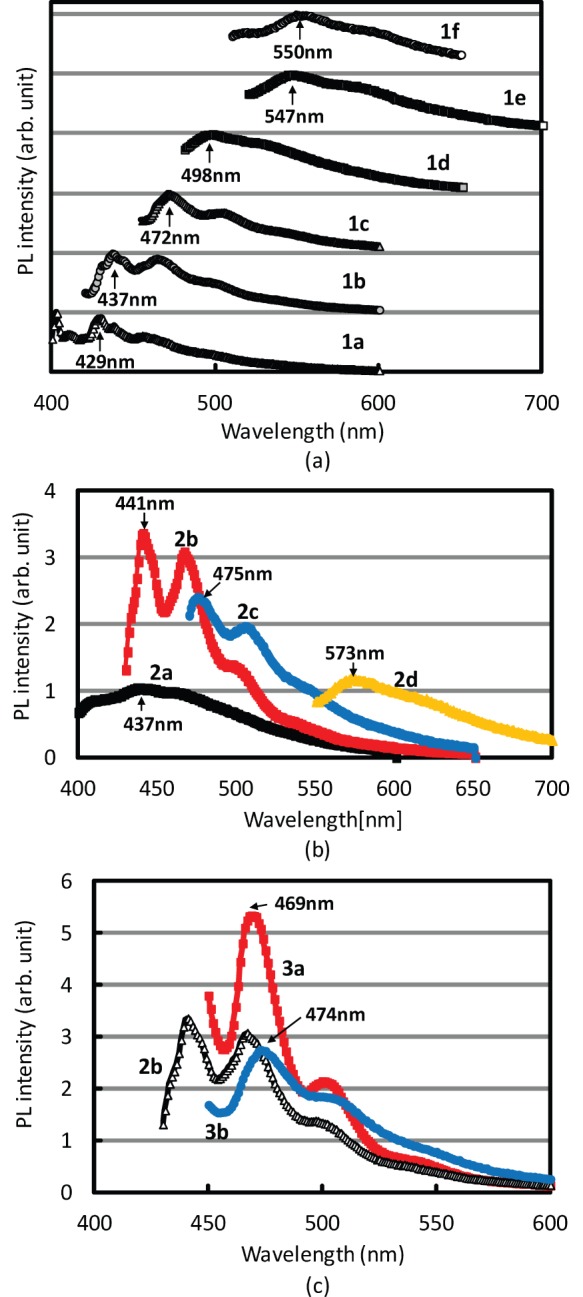
PL spectra of coal tar pitch solutions. (a) **1a**–**1f** [excitation wavelength: 379 nm (**1a**), 405 nm (**1b**), 437 nm (**1c**), 454 nm (**1d**), 495 nm (**1e**), and 489 nm (**1f)**, (b) **2a–2d** [excitation wavelength: 364 nm (**2a**), 408 nm (**2b**), 440 nm (**2c**), and 523 nm (**2d)**, and (c) **3a**, **3b** [excitation wavelength: 374 nm (**3a**) and 374 nm (**3b**)]. Solutions **2a**–**2d** and **3a**, **3b** were obtained by PTLC separation.

Solutions **1a**–**1f**, **2a**–**2d**, **3a**, and **3b** were diluted in 1∶100 ratio (by weight), and the PL spectra were recorded. The normalized PL spectra for solutions **1a**–**1f** are shown in Fig. 1(a). The peak patterns in the PL spectra differed, indicating that the polar components of coal tar pitch, which have extended π-conjugation, dissolved to a greater extent in polar solvents. This is because that a black solids were obtained at amounts of 2.6 g for cyclohexane, 2.6 g for TBME, 3.2 g for CPME, 3.8 g for benzene, 3.8 g for THF, and 3.7 g for CHCl_3_ from the coal tar pitch (5.0 g). The excitation wavelengths were fixed at the peak wavelength of the measured photoluminescence excitation (PLE) spectra of the coal tar pitch solutions: 379, 405, 437, 454, 495, and 489 nm for **1a**, **1b**, **1c**, **1d**, **1e**, and **1f**, respectively. The PL wavelength was the shortest (429 nm) for solution **1a** (cyclohexane) and the longest (550 nm) for solution **1f** (CHCl_3_). Most importantly, the PL wavelength of the coal tar pitch solution could be controlled by appropriate choice of the solvent, similar to a previously reported case in which the PL spectra in two different solvents were compared [17], [28].

**Figure 2:**
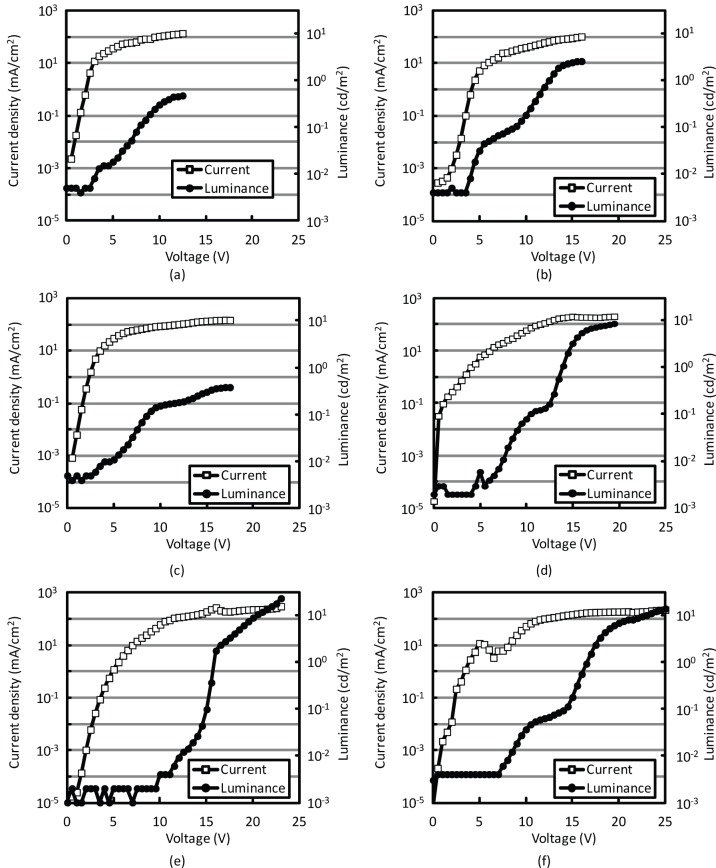
Current density-voltage-luminance characteristics of OLEDs with emissive layers prepared using coal tar pitch solutions. **1a** (cyclohexane), **1b** (TMBE), **1c** (CPME), **1d** (benzene), **1e** (THF), and **1f** (CHCl_3_).

**Figure 3:**
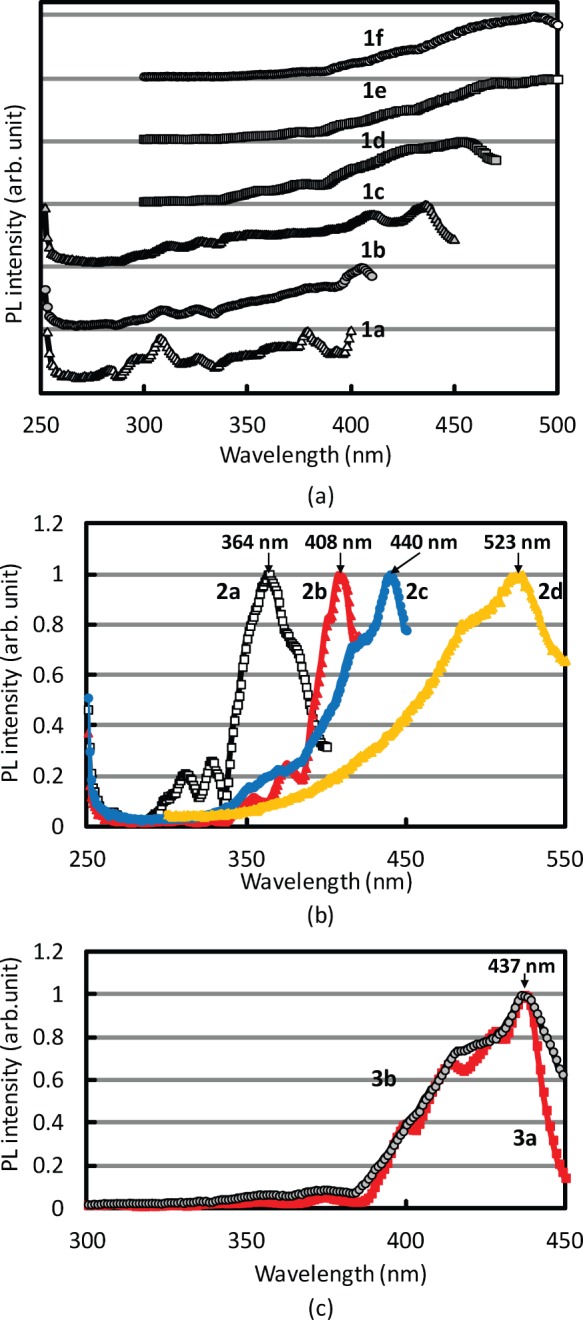
PLE spectra of coal tar pitch solutions. (a) **1a–1f**, (b) **2a–2d,** and (c) **3a, 3b**.

Figure 1(b) shows the PL spectra of the preparative thin-layer chromatography (PTLC)-separated coal tar pitch solutions **2a**–**2d**, whose concentration is 100 µg/mL. Here, we chose THF was a solvent for the further separation technique by using the PTLC process. This is because that the highest luminance was achieved when THF was used, as shown in Fig. 2. The less polar (high-*R_f_*) components had shorter peak wavelengths, indicating that the PL spectrum could be controlled by optimizing the polarities of the stationary and mobile phases used in PTLC. Among **2a**–**2d**, the **2b** solution showed the highest PL intensity at 441 nm, with smaller peaks at 465, 497, and 537 nm. The PL spectra of coal tar pitch solutions **3a** and **3b** are shown in Fig. 1(c). The peak wavelengths were 469 nm for **3a** and 474 nm for **3b**, and the peak emission at 441 nm, which appeared for the solution **2b**, was disappeared after the PTLC process (**3a** and **3b**). This is because that the solutions **3a** and **3b** showed the peak PLE spectrum at 437 nm (Fig. 3(c)), and this indicates that the emission at 471 nm was absorbed by other molecules in the PTLC-separated coal tar pitch in **3a** and **3b**. The PL intensity was relatively higher in the case of solution **3a** than in the case of solution **3b**. These observations led us to conclude that it is advantageous to separate solution **2b** into smaller fractions on the basis of polarity and that multiple rounds of PTLC can help improve the PL intensity of the coal tar pitch solution. In addition, the raw coal tar pitch contains many kinds of emissive molecules, therefore the composition and the molecular structures of extracted coal tar pitch are different each other owing to the *Rf* value during the PTLC process. Therefore, the PL spectra of extracted coal tar pitch (**2a**–**2d**, **3a**, **3b**) are different, as shown in Figs. 1(b) and (c).

**Figure 4:**
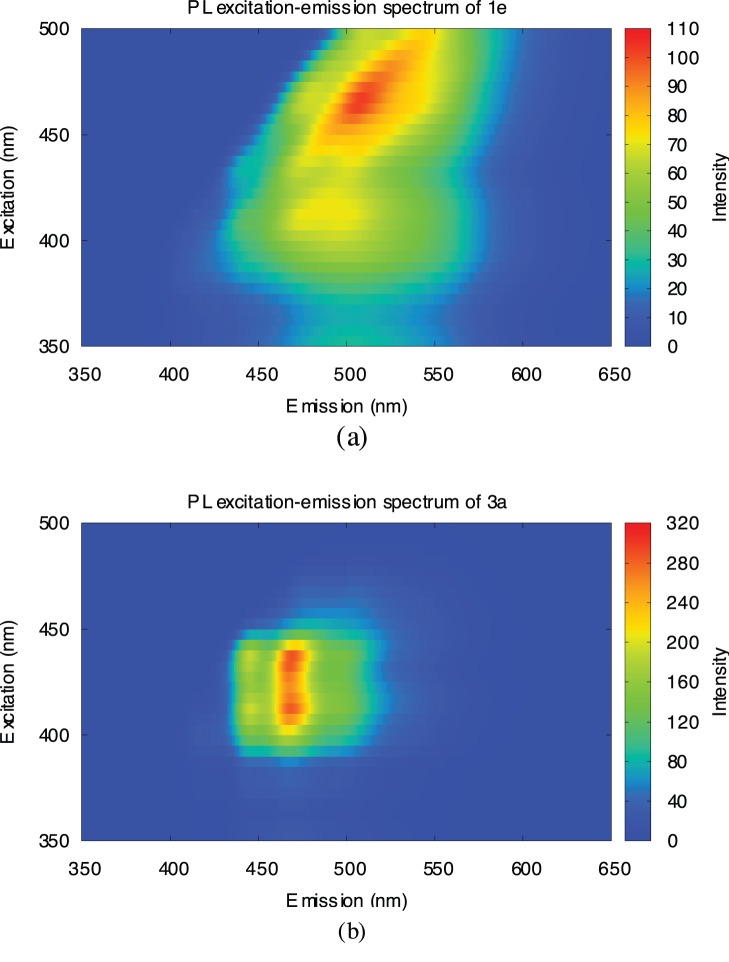
Excitation-emission matrix spectra. (a) **1e** and (b) **3a** with normalized intensities.

The coal-pitch solutions were also analyzed by excitation-emission matrix (EEM) spectroscopy, which is frequently used for the analyses of PAH-containing oil [29], PAH impurities in water [30], and nanocarbon materials [19], [20], [31]–[34]. The excitation wavelength ranged from 350 nm to 500 nm, and the emission wavelength ranged from 350 nm to 650 nm. The EEM spectrum of the tetrahydrofurane (THF) solution of coal tar pitch (Fig. 4(a)) showed a longer shift in the emission wavelength, in line with the longer excitation wavelength, indicating the presence of numerous emissive components in coal tar pitch. As a result, the emission maxima covered a broad range of wavelengths, 450–550 nm. In contrast, the EEM spectrum of the THF solution of PTLC-separated coal tar pitch (Fig. 4(b)) showed a narrower distribution of emission wavelengths, 450–500 nm. These spectral observations indicated that the emissive components in coal tar pitch could be efficiently extracted by two rounds of chromatographic separation.

Owing to their high absorbance, solutions **1a**–**1f**, **2a**–**2d**, **3a**, and **3b** were diluted in 1∶100 weight ratio using the same solvent. The resulting solutions were filled in 10-mm-long quartz glass cells, and PLE spectra were recorded [monitoring wavelength: 429 nm (**1a**), 437 nm (**1b**), 472 nm (**1c**), 500 nm (**1d**), 547 nm (**1e**), 550 nm (**1f**), 437 nm (**2a**), 441 nm (**2b**), 475 nm (**2c**), 573 nm (**2d**), 469 nm (**3a**), and 474 nm (**3b**]. The monitoring wavelength was defined as the measured PL peak wavelength. The obtained PLE spectra are summarized in Figures 3(a) (**1a**–**1f**), 3(b) (**2a**–**2d**), and 3(c) (**3a** and **3b**). The maximum absorption wavelengths were used as the excitation wavelengths for the PL spectra in Figs. 1(a)–(c). The PLE spectrum can be also explained the polar components of coal tar pitch, as discussed in the Fig. 1(a). The higher polar components caused the longer excitation wavelength, resulting in the longer emission wavelength.

Each coal tar pitch solution was spin-coated on the surface of an ITO-glass substrate covered with poly(3,4-ethylenedioxythiophene):poly(styrenesulfonate) (PEDOT:PSS), and Li/Al electrodes were deposited onto the resulting coal tar pitch surface. Figures 2(a)–(f) show the current density-voltage-luminance characteristics of OLEDs with coal tar pitch (emissive) layers formed from solutions **1a** (cyclohexane), **1b** (*tert*-butyl methyl ether, TBME), **1c** (cyclopentyl methyl ether, CPME), **1d** (benzene), **1e** (THF), and **1f** (CHCl_3_). The current densities of the OLEDs were 36.4 mA/cm^2^ (**1a**), 5.14 mA/cm^2^ (**1b**), 29.8 mA/cm^2^ (**1c**), 5.95 mA/cm^2^ (**1d**), 1.15 mA/cm^2^ (**1e**), and 11.6 mA/cm^2^ (**1f**) when the applied voltage was 5 V. Therefore, the highest and lowest current densities were observed for the OLEDs in which the emissive layers were formed from solutions **1a** and **1e**, respectively, and the highest luminance of 22.4 cd/m^2^ was realized when the THF solution of **1e** was used. In a conventional OLED, holes and electrons are injected from the ITO and Al electrodes, respectively, and the luminance efficiency can be enhanced by improving the carrier balance in the emissive layer. In the present study, the luminance tended to increase with a decrease in the current density when using solutions **1a**, **1e**, and **1f**, because of the carrier balance in the emissive layer. The highest luminance corresponds to the lowest current density in the case of solution **1e**; therefore, this indicates that the excess carriers prevent the efficient carrier recombination in the coal tar pitch layer, resulting in the highest luminance for the device with **1e** solution. In particular, the emissive molecules could be extracted by dissolution in THF.

**Figure 5:**
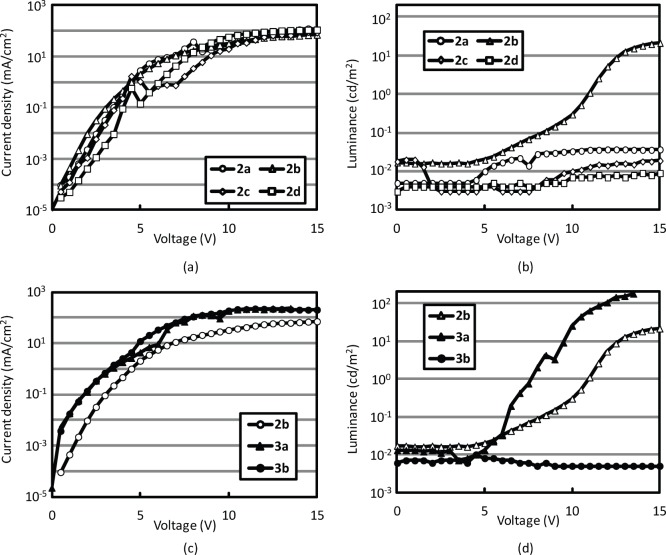
Current density-voltage (a) and luminance-voltage (b) characteristics of devices with coal tar pitch solutions 2a, 2b, 2c, and 2d. Current density-voltage (c) and luminance-voltage (d) characteristics of devices with coal tar pitch solutions **2b**, **3a**, and **3b**.

Figures 5(a) and (b) show the current density-voltage and luminance-voltage characteristics of OLEDs in which the emissive layers were prepared from solutions **2a**, **2b**, **2c**, and **2d**. Since the luminance characteristics were influenced by the *R_f_* value, we could conclude that all the fractions obtained from **1e**, except for **2b**, contained very small amounts of emissive molecules. The maximum luminance of the OLED prepared using solution **2b** was 19.7 cd/m^2^, since the maximum PL intensity was observed for this solution, as shown in Fig. 1(b). The maximum luminance of the device with solution **2b** was almost the same as that of the reference device with solution **1e**; however, the drive voltage was drastically reduced, as shown in Figs. 2(e) and 5(b), probably because the polar component in solution **1e** was removed by PTLC. The current density-voltage and luminance-voltage characteristics of the OLEDs prepared using solutions **2b**, **3a**, and **3b** are shown in Figs. 5(c) and (d). The current density and luminance were higher for the device prepared using solution **3a** (maximum luminance: 176 cd/m^2^ at 13.5 V) than for the device prepared with solution **2b**. This result indicated that the second PTLC separation using CH_2_Cl_2_/hexane as the eluent caused the additional removal of some high-polarity (high-*R_f_*) component, thereby resulting in improved device performance.

**Figure 6:**
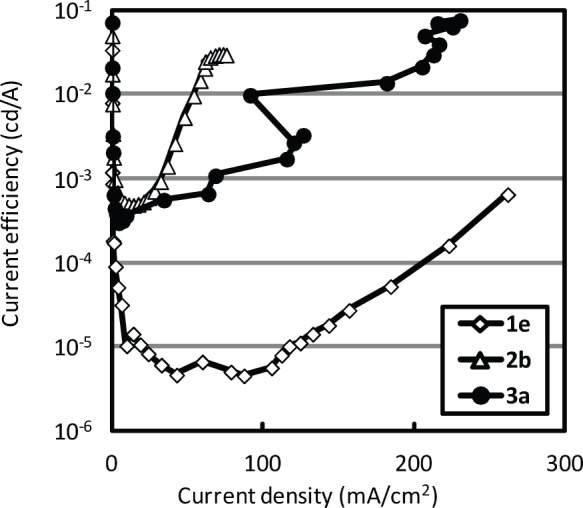
Current efficiency-current density characteristics of OLEDs with coal tar pitch solutions. **1e**, **2b**, and **3a.**

Figure 6 shows the current efficiency-current density characteristics of OLEDs prepared using solutions **1e**, **2b**, and **3a**. The maximum current efficiency of the device with solution **1e** was 6.5×10^−4^ cd/A, while the maximum efficiencies of the devices prepared with solutions **2b** and **3a** were 3.0×10^−2^ cd/A and 7.6×10^−2^ cd/A, respectively. The current efficiency, an important parameter deciding the OLED performance, was improved by the PL quantum efficiency and the carrier balance in the emissive layer. As shown in Fig. 2(c), the current density of the device prepared with solution **3a** was approximately 10 times higher than that of the device prepared with solution **2b**. In this device structure, the electron density in the emissive layer is considered to be much higher than the hole density. The high current density disturbed the carrier balance in the emissive layer. However, the luminance was improved when using the coal tar pitch solution **3a**. This indicated that the difference in luminance was caused by the PL quantum yield of the coal tar pitch solution and that the PL quantum efficiency of the solution was improved by PTLC separation. The highest luminance was observed in the case of the OLED fabricated using solution **3a**.

### Conclusion

In conclusion, we demonstrated the photophysical properties of coal tar pitch solutions prepared by simple addition and subsequent separation by PTLC. The PL spectra of coal tar pitch extracted with different solvents showed that the emission wavelength was longer when using a polar solvent. Further, the solution containing a large amount of high-*Rf* (less polar) components had a shorter emission wavelength. The emissionexcitation matrix spectra showed that the emission range of the coal tar pitch solution narrowed after PTLC separation, indicating the relatively low concentration of emissive components. The characteristics of the OLEDs prepared using coal tar pitch as an emissive layer could be controlled by modifying the solution preparation method. The best luminance of 176 cd/m^2^ at 13.5 V was achieved in the case of the OLED fabricated using solution **3a**, which was obtained by two rounds of PTLC separation of **1e**. The current efficiency-density characteristics for the obtained OLED device showed that PTLC separation led to improved PL quantum yield. These results suggest that coal tar pitch is an inexpensive organic material that is suitable for use in OLEDs. Further research aimed at improving the OLED properties and analyzing the feasibility of using coal tar pitch in other types of organic devices is underway.

## Materials and Methods

Various solutions of coal tar pitch were prepared as follows. Simple addition of coal tar pitch, whose softening point is around 90°C, in conventional organic solvents such as cyclohexane, TBME, CPME, benzene, THF, and CHCl_3_ yielded deep-brown suspensions, which were then filtered to obtain homogeneous solutions (referred to as **1a**, **1b**, **1c**, **1d**, **1e**, and **1f**, respectively). The solubility of coal tar pitch in each solvent was estimated in terms of the amount of coal tar pitch used: 52%, cyclohexane, (**1a**); 52%, TBME (**1b**); 64%, CPME (**1c**); 76%, benzene (**1d**); 76%, THF (**1e**); and 74%, CHCl_3_ (**1f**).

A 100-mL Erlenmeyer flask equipped with a magnetic stirring bar was charged with coal tar pitch (5.0 g) and the desired solvent (100 mL, cyclohexane for **1a**, TBME for **1b**, CPME for **1c**, benzene for **1d**, THF for **1e**, and CHCl_3_ for **1f**). The resulting mixture was stirred at room temperature for 4.5 h. After removal of the coal tar pitch residue by filtration, the filtrate was evaporated under reduced pressure to give a black solid (2.6 g for cyclohexane, 2.6 g for TBME, 3.2 g for CPME, 3.8 g for benzene, 3.8 g for THF, 3.7 g for CHCl_3_). This black solid was dissolved in the desired solvent to obtain solutions **1a**–**1f** with the appropriate concentrations (2.6 mg/mL for cyclohexane, 2.6 mg/mL for TBME, 3.2 mg/mL for CPME, 10 mg/mL for benzene, 10 mg/mL for THF, and 10 mg/mL for CHCl_3_).

A given portion (5 mL, 50 mg, 10 mg/mL) of **1e** was separated into four fractions with *R_f_* values of 1.0–0.75 for **2a**, 0.75–0.5 for **2b**, 0.5–0.25 for **2c**, and 0.25–0 for **2d**, by PTLC using hexane/Et_2_O (4∶1) as the eluent. All the four fractions were extracted with THF, and the resulting suspensions were filtered through a glass filter to remove silica gel. Volatiles were removed under vacuum to obtain black solids (*R_f_* 1.0–0.75, 0.0297 g; *R_f_* 0.75–0.5, 0.0712 g; *R_f_* 0.5–0.25, 0.0481 g; *R_f_* 0.Fig. 5–0, 0.1114 g). These solids were dissolved in appropriate amounts of THF to prepare solutions **2a**–**2d** with concentrations of 10 mg/mL.

A portion (8 mL, 80 mg, 10 mg/mL) of **2b** (*R_f_* 0.75–0.5) was further separated on a PTLC plate with a CH_2_Cl_2_/hexane (1∶3) eluent [35], [36]. The yellow and dark-yellow bands at around *R_f_* = 0.7 were extracted with THF, and the resulting suspensions were filtered through a glass filter. Volatiles were removed under vacuum to obtain black solids (yellow fraction: 0.0263 g, dark-yellow fraction: 0.0096 g). These solids were dissolved in appropriate amounts of THF to obtain two 10 mg/mL solutions, **3a** and **3b**. Silica gel PTLC plates were prepared in-house using Wakogel B-5F (Wako Pure Chemical Industries, Ltd.).

PL and PLE spectra were measured using a spectrofluorometer (FP-6200, JASCO). Coal pitch (softening point: around 90°C, which is commercially available from oil or coal companies all over the world, was used in this study.

A fabrication process of OLED is as follows, and it is well-known standard process for the OLED field [3]–[7]. A 150-nm-thick indium tin oxide (ITO) layer was sputtered on a glass substrate. The ITO-coated glass substrate was ultrasonically cleaned in organic solvent/deionized water and subjected to ultraviolet ozone cleaning for 20 min. PEDOT:PSS (P AI 4083, H.C. Stark) was spin-coated onto the ITO layer (2000 rpm, 60 s) in nitrogen atmosphere. The thickness of the PEDOT:PSS layer was estimated to be 70 nm by using a surface profiler system (Alpha Step IQ, KLA Tencor). After annealing at 140°C for 10 min, the coal tar pitch solution was spin-coated onto the PEDOT/PSS layer (2000 rpm, 60 s). The thicknesses of the coal tar pitch layers were 86 nm for **1a**, 58 nm for **1b**, 69 nm for **1c**, 82 nm for **1d**, 98 nm for **1e**, and 104 nm for **1f**, as estimated using the surface profiler system. Finally, LiF (1 nm, 0.05 nm/s) and Al (120 nm, 0.1–0.3 nm/s) were deposited onto the coal tar pitch layer. The deposition rates of LiF and Al were measured with a quartz crystal monitor. Each glass substrate had four OLEDs with an area of 9 mm^2^. The current density-voltage-luminance characteristics were measured using a DC voltage current source/monitor (6241, ADCMT) and a luminance meter (LS-100, Konicaminolta).
